# Supplementary material for: Impact of demographic factors and HIV status on Hepatitis B vaccination adherence and completion rate among high-risk populations in Lagos State, Nigeria
Source: PLoS One. 2026 Mar 4;21(3):e0321434. doi: 10.1371/journal.pone.0321434 (PMC12959706; doi:10.1371/journal.pone.0321434)
Supplement: Attachment — Submitted filename: Response_to_Reviewers.docx [file pone.0321434.s003.docx]

**Response to the Academic Editor’s and Reviewers’ Comments**

**Manuscript Title:** Impact of demographic factors and HIV status on Hepatitis B vaccination adherence and completion rate among high-risk populations in Lagos State, Nigeria
**Manuscript ID:** PONE-D-25-10988

Dear Dr. Blackard,

We sincerely thank you and the reviewers for the constructive and insightful comments on our manuscript. Also, we are grateful for the opportunity to revise and improve the paper. We have carefully addressed all editorial and reviewer comments. We believe the revised manuscript has been substantially improved accordingly. We confirm that all required changes have been included in the revised manuscript. Both the tracked-changes version and an unmarked clean copy have been uploaded as requested. Our point-by-point response to the journal requirements, additional editor’s and reviewers’ comments are provided below.

**RESPONSES TO JOURNAL REQUIREMENTS**

**1. PLOS ONE Style and Formatting**

**Response:**
We have thoroughly reviewed the manuscript and ensured full compliance with PLOS ONE style requirements. This includes the formatting, file naming conventions, and structure, in line with the official PLOS ONE templates, detailing journal specifications.

**2. Funding Statement Clarification**

**Response:**
We appreciate the clarification regarding funding disclosures. In line with PLOS ONE policy, we have removed all funding-related text from the body of the manuscript as well as the Acknowledgments section. The Funding Statement has been updated and made available through the cover letter. It now accurately reflects the funding context of the vaccination program which gave rise to the data used in this study. We ensured total transparency regarding the absence of direct research funding.

**3. Data Availability Statement**

**Response:**
We acknowledge PLOS ONE’s open data policy and revised the data availability statement section of the manuscript. We have decided to make the de-identified version of the dataset publicly available, and the DOI has been included in the revised Data Availability Statement for easy access. Below is the revised statement: *“The secondary data used in this study was generated from the community-based vaccination program implemented by CPHI. The data are available at the OSS clinic of CPHI, and publicly available at Figshare: https://doi.org/10.6084/m9.figshare.30998749. Also, all data related requests or inquiries can be sent to info@cphinigeria.org or to the corresponding author.”*

**4. Ethics Statement Placement**

**Response:**
The Ethics Statement has been moved exclusively to the Methods section as required. Also, the ethics approval details are clearly and correctly presented in accordance with PLOS ONE guidelines.

**5. Supporting Information Captions and review of reference list**

**Response:**
We do not have any supporting document/information referenced in the manuscript. On the other hand, we thoroughly reviewed the list of references and corrected the ‘referencing style’ to match PLOS ONE guidelines. Also, we noticed that reference numbers 5 and 22 were duplicates, so we corrected it by replacing number 22 with the appropriate article reference. No redacted article was cited in the manuscript.

**RESPONSES TO ADDITIONAL EDITOR COMMENTS**

**Additional Editor’s Comment:**

“This is a cross-sectional study of HBV vaccination rates conducted in Lagos, Nigeria. The methods and results are described well, although the findings are not particularly surprising. In addition to the comments raised by the two reviewers, additional clarifications below would strengthen the manuscript further:

- The introduction must include information about the availability of HBV vaccination in the public and private sectors across Nigeria as well as any associated cost.  Similarly, what are the nationwide policies/guidelines on HBV vaccination?
- Are HBV medications available in the public and private sectors?
- It is unclear why data prior to 2021 were not included.”

**Response:**

- Regarding the availability and Cost of HBV Vaccination in Nigeria, we have revised the Introduction to explicitly describe the availability of hepatitis B vaccination across public and private healthcare facilities in Nigeria. Although economic factors are not part of the demographic factors assessed in this study, we also clarified that, HBV vaccination is provided free of charge to infants in public primary healthcare facilities as part of Nigeria’s National Program on Immunization (NPI). Outside routine infant immunization, HBV vaccination for adolescents and adults is commonly fee-based, particularly in private healthcare facilities. However, costs in the private sector vary from provider type to another, and from one geographic location to another. This is potentially a financial barrier to HBV vaccine uptake, but this was not part of the demographic factors assessed in this study. Regarding the national policies and guidelines on HBV vaccination, we have expanded the Introduction to mention that all HBV vaccination programs in Nigeria are guided by the National Program on Immunization (NPI) guidelines, the National Policy on Viral Hepatitis, and strict alignment with WHO hepatitis elimination targets. We clearly mentioned administration of HBV vaccine using the 0-1-6 vaccination schedule as provided for by the various guidelines.
- Regarding availability of HBV Medications, we have clarified in the revised manuscript that antiviral medications for chronic hepatitis B management are available in both public and private healthcare facilities in Nigeria. However, access, cost, and availability may vary by levels of facility and geographic location.
- Regarding the reason data prior to 2021 were not included, we have clarified that the HBV vaccination program fully commenced in 2021 at the study site. The study relied on routinely collected programmatic data that were systematically standardized and complete only from 2021 onward. After due programmatic harmonization and improved data quality assurance processes, we used data from 2021 to 2024, and inclusion of data prior to 2021 would have compromised internal validity and overall data quality. This rationale is now clearly stated in the Methods Section, to enhance transparency and methodological rigor.

**RESPONSES TO REVIEWER-1 COMMENTS**

We thank the reviewer for sharing the ScreenIT report with us. We have carefully read the report and below are our responses to all the sections flagged for potential issues.

**Rigor Section**

- **Flow charts and attrition:**

**Response:** Based on our study design a flow chart is not required, same for sentences about attrition. So, we would like to proceed with the manuscript without a flow chart.

- **Randomization and Blinding**

**Response:** This study is a retrospective observational analysis of routinely collected vaccination records. No experimental interventions were performed; therefore, no randomization or blinding procedures were applicable.

- **Power Analysis**

**Response:** Since the study was a retrospective analysis of routinely collected secondary data. a formal power or sample size calculation was not performed. Instead, we included all patient records for individuals who accessed vaccination services during the predefined study period. The sample therefore represents a survey of all eligible participants, rather than a sampled subset. This is an acceptable approach for the evaluation of retrospective study data. So, the large number of observations often associated with studies on secondary data provided sufficient precision for descriptive and comparative analyses.

**Resources Section**

- **Software and Algorithms**

**Response:** RRIDs were not added for Microsoft Excel or SPSS because these are standard, widely used software tools. Also, RRIDs are not specifically required by the PLOS ONE journal’s author guidelines. So, we would like to proceed with the manuscript without adding any RRIDs.

**Transparency Section**

- **Open code**

**Response:** This study did not involve the use of custom-written analysis code. All analyses were conducted using Microsoft Excel and IBM SPSS Statistics. As such, there is no analysis code available or developed for public sharing.

- **Open data**

**Response:** The dataset analyzed in this study is essentially a routinely collected health service data. However, de-identified data have been deposited in a public repository, and a dedicated DOI has been made available in the data availability section of this manuscript. Additionally, institutional contact was made available to address all source data related inquiries or requests.

- **Self-acknowledged limitations**

**Response:** We can confirm that there is a section explicitly discussing the study limitations in this manuscript. This section addressed limitations with the scope of data and generalizability. We also recommended future studies that could potentially close the gaps or limitations of this study.

**RESPONSES TO REVIEWER – #2 COMMENTS**

We sincerely thank Reviewer – #2 for the thoughtful review of our manuscript and for the positive assessment of the study’s design, analysis, and conclusions. We are grateful for the constructive suggestions, all of which have been carefully addressed. Our detailed point-by-point responses are provided below.

**Reviewer comment 1:**

“It is assumed that the vaccination schedule followed in this study was 0-1-6 and it is important to include these details in the methodology section.”

**Response:**

We agree with you on this important suggestion. We have now explicitly stated in the Methods section that the hepatitis B vaccination schedule in this study followed the standard 0–1–6 months schedule, in line with national and WHO-recommended practices in Nigeria. This clarification has been added to improve methodological transparency. See line 133 of the revised manuscript.

**Reviewer comment 2:**

“In line No.189, the age range mentioned is 5–29 years and this typo may be corrected.”

**Response:**

Thank you for noting this error. The age range has now been corrected to reflect the accurate study population age of “25 – 29”. The typographical error has been fixed.

**Reviewer comment 3:**

“In line 322, is it “holistic” or “wholistic”?”

**Response:**

We appreciate this observation. The word has been corrected to “holistic,” which is the standard and widely accepted spelling in scientific writing.

**Reviewer comment 4:**

“In futurology, maybe the authors could consider including antibody titre post vaccination, and this will assess the respondent rate.”

**Response:**

We thank the reviewer for this valuable forward-looking suggestion. We have now included this point in the Limitations and Future Research section. We noted that the inclusion of post-vaccination antibody titre (anti-HBs) assessment in future studies would help to evaluate immunological response and sero-protection levels. Although such laboratory measurements were not within the scope of the current retrospective study, we acknowledge that it represents an important area for future research. Refer to line 344 of the revised manuscript.

**Closing Statement**

Once again, we thank the academic editor and the reviewers for the constructive feedback, which has significantly strengthened the manuscript. We believe the revised version now fully meets PLOS ONE’s publication criteria and addresses all editorial and reviewer concerns.

We look forward to your kind consideration of our revised submission.

Yours Sincerely,

**Ojore Godday Aghedo**
(On behalf of all authors)
